# The Stoichiometry of Isoquercitrin Complex with Iron or Copper Is Highly Dependent on Experimental Conditions

**DOI:** 10.3390/nu9111193

**Published:** 2017-10-30

**Authors:** Maria Carmen Catapano, Václav Tvrdý, Jana Karlíčková, Thomas Migkos, Kateřina Valentová, Vladimír Křen, Přemysl Mladěnka

**Affiliations:** 1 Department of Pharmacology and Toxicology, Faculty of Pharmacy in Hradec Králové, Charles University, Heyrovského 1203, 500 05 Hradec Králové, Czech Republic; catapanm@faf.cuni.cz (M.C.C.); tvrdyvac@faf.cuni.cz (V.T.); migkost@faf.cuni.cz (T.M.); 2 Department of Pharmaceutical Botany, Faculty of Pharmacy in Hradec Králové, Charles University, Heyrovského 1203, 500 05 Hradec Králové, Czech Republic; karlickova@faf.cuni.cz; 3 Laboratory of Biotransformation, Institute of Microbiology, Czech Academy of Sciences, Vídeňská 1083, 142 20 Prague, Czech Republic; kata.valentova@email.cz (K.V.); kren@biomed.cas.cz (V.K.)

**Keywords:** chelator, copper, iron, quercetin-3-*O*-β-glucopyranoside, Job’s method, stoichiometry, reduction

## Abstract

Interaction of flavonoids with transition metals can be partially responsible for their impact on humans. Stoichiometry of the iron/copper complex with a flavonoid glycoside isoquercitrin, a frequent component of food supplements, was assessed using competitive and non-competitive methods in four (patho)physiologically-relevant pH values (4.5. 5.5, 6.8, and 7.5). Isoquercitrin chelated all tested ions (Fe^2+^, Fe^3+^, Cu^2+^, and Cu^+^) but its affinity for Cu^+^ ions proved to be very low. In general, the chelation potency dropped with pH lowering. Metal complexes of 1:1 stoichiometry were mostly formed, however, they were not stable and the stoichiometry changed depending on conditions. Isoquercitrin was able to reduce both Cu^2+^ and Fe^3+^ ions at low ratios, but its reducing potential was diminished at higher ratios (isoquercitrin to metal) due to the metal chelation. In conclusion, this study emphasizes the need of using multiple different methods for the assessment of chelation potential in moderately-active metal chelators, like flavonoids.

## 1. Introduction

**Figure 1:**
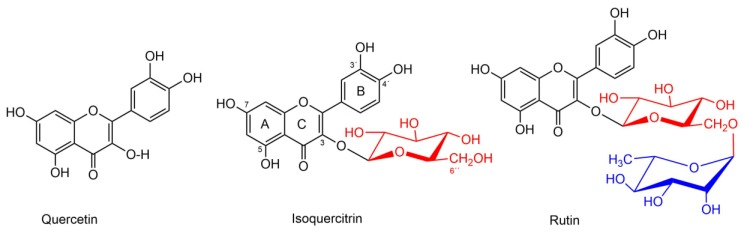
Chemical structures of quercetin and its glycosides isoquercitrin and rutin. The differences are shown in color and consist of the attached sugar moiety at C-3 of quercetin: rutinose (l-α rhamnopyranosyl(1-6)β-d-glucopyranose) in the case of rutin, while β-d-glucopyranose in the case of isoquercitrin.

Isoquercitrin (quercetin-3-*O*-β-d-glucopyranoside (Figure 1) is one of the most common glycosides of quercetin occurring in various fruits and vegetables, and currently one of the most popular flavonoids used in various food supplements with a large number of potential health benefits [1,2]. A wide range of biological effects were reported for isoquercitrin, including in vivo antioxidant [3], anti-inflammatory [4], anticancer [5], cardioprotective [6], antidiabetic [7], anti-allergic [8], and neuropharmacological [9] activities. Moreover, bioavailability of quercetin was found to be higher when using isoquercitrin as its source, compared to the aglycone itself [10]. Some studies even reported intact isoquercitrin in rat plasma [11,12,13,14] and rat brain [15] after consumption of plant extracts rich in this flavonoid. Although isoquercitrin is often found in medicinal plants (St. John’s wort), fruits (apples, berries), vegetables (onion, garlic), and in plant-derived beverages (tea, wine) [1,16], the content of isoquercitrin in plant material is low and isolation techniques are, thus, impracticable [15]. Hence, this compound had been available only as an analytical standard and, therefore, it was also scarcely tested for biological activity compared to other flavonoids. However, an efficient biocatalytic method for the production of pure isoquercitrin from rutin (see Figure 1 for the structural relationship with isoquercitrin) was developed [17] based on the use of α-l-rhamnosidase [18] from *Aspergillus terreus*, heterologously expressed in *Pichia pastoris*, which affords isoquercitrin in very high yields and purity (97–99.9%). Interestingly, despite numerous studies on antioxidant activity of isoquercitrin [3,5,19,20,21], its chelation potential was not studied up to date, with the exception of a complex study on aluminum and further papers reporting Fe^2+^ chelation without any detailed data on the stoichiometry [22,23,24]. Additionally, many controversies have been raised, in particular, related to pro-oxidative properties of flavonoids [25,26]. These can result from interactions with transition metals, such as iron and copper. Tight chelation of these metals is considered to have an antioxidant effect while their reduction can facilitate the Fenton reaction and, hence, be pro-oxidative. For example, quercetin and rutin were found to reduce lipid peroxidation by chelation of ferrous ions [27], but the relationship between flavonoids and anti/pro-oxidation seems to be rather dose-dependent [25]. Additionally, there are some studies supporting the chelation potential of flavonoids toward copper, and also their ability to reduce cupric ions [28,29,30,31].

The aim of the present work was to characterize the interactions of pure isoquercitrin with the transition metals iron and copper at pathophysiologically-relevant pH. The selection of pH ranging from 4.5 to 7.5 was based on the assumption that the physiologically most relevant interactions can take place in the gastrointestinal tract where the pH raises from the duodenum to jejunum.

## 2. Materials and Methods

### 2.1. Chemicals, Solutions, and Equipment

Isoquercitrin (Figure 1, purity 99.82%) was prepared by enzymatic trimming of rutin using α-l-rhamnosidase from *Aspergillus terreus* heterologously expressed in *Pichia pastoris* [17]. 3-(2-Pyridyl)-5,6-diphenyl-1,2,4-triazinedisulfonic acid sodium salt (ferrozine), FeSO_4_ · 7 H_2_O, FeCl_3_ · 6 H_2_O, ferric tartrate (Fe_2_(C_4_H_4_O_6_)_3_), hydroxylamine hydrochloride (HA), CuSO_4_ · 5 H_2_O, CuCl, disodium bathocuproinedisulfonate (BCS), dimethylsulfoxide (DMSO) and chemicals for buffers were purchased from Sigma-Aldrich Inc. (Darmstadt, Germany) and methanol from J.T. Baker (Avantor Performance Materials, Inc., Center Valley, PA, USA). Ultrapure water (Milli-Q RG, Merck Millipore, Burlington, MA, USA) was used throughout this study. Stock solutions of ferrozine, Cu/Fe salts, BCS (all 5 mM), and HA (100 mM) were prepared in water, with the exception of CuCl (5 mM), which was dissolved in the aqueous solution of 0.1 M HCl and 1 M NaCl. All experiments for the stoichiometry determination were performed in semi-micro polystyrene or ultraviolet-transparent cuvettes (BrandTech Scientific Inc., Essex, CT, USA) and the absorbance was measured by Helios Gamma equipped with VisionLite 2.2 software (ThermoFisher Scientific Inc., Waltham, MA, USA), while competitive experiments were performed in 96-well microplates (BRAND GmbH + CO KG, Wertheim, Germany) with spectrophotometer Synergy HT Multi-Detection Microplate Reader (BioTec Instruments, Inc., Winooski, VT, USA).

### 2.2. PH Conditions

Experiments were performed at four (patho)physiologically-relevant pH values (4.5. 5.5, 6.8, and 7.5). Acetate buffers (15 mM of sodium acetate and 27.3 and 2.7 mM of acetic acid, respectively) were used for the two lower pH values, whereas HEPES (4-(2-hydroxyethyl)-1-piperazineethanesulfonic acid) buffers (15 mM of sodium HEPES and 71.7 and 14.3 mM of HEPES, respectively) were used for pH 6.8 and 7.5. Based on our previous experiments [28], chloride was used for ferric ion solutions at pH 4.5 and 5.5, while tartrate was used at pH 6.8 and 7.5. As the oxidation of Fe^2+^ accelerates significantly at pH 7.5, HA in the final concentration of 5 mM was added to this buffer to avoid oxidation. In the case of Cu^+^ ions, HA was added in same concentration to all buffers to retain the ion in the lower valence.

### 2.3. Assessment of Iron/Copper Complex Stoichiometry

The assessment of the stoichiometry was done according to the previously established protocol [32].

#### 2.3.1. Complex Formation

Firstly, we checked whether the metal is forming a complex with isoquercitrin. Based on the previous studies in the field [33,34], clear shift(s) in the absorbance maximum (maxima) of a flavonoid after addition of a metal ion is considered to be due to the formation of the complex. In flavonoids, the absorbance bands corresponding to the rings B + C (see Figure 1) lie in the range 320–385 nm and display a bathochromic shift (to higher wavelengths) after a complex with a metal ion is formed. Hence, in our study, a methanolic solution of isoquercitrin was mixed with a water solution of metal ions for 1 min at different molar concentration ratios ranging generally from 1:4 to 1:50 (isoquercitrin:metal) at all tested pH conditions. The concentration of isoquercitrin ranged from 33 to 83 μM while that of the metal was always 500 μM. These measurements were also performed without metal ions and used for the calculation of molar absorption coefficients, which were needed for further analysis (see Section 2.3.3). Absorption spectra *vs.* the blank were measured. The blank for pure isoquercitrin and ferrous complex measurements was composed of buffer, methanol, and water in a 1:1:1 ratio, while, for the ferric complex, the composition was the same with the exception that it also contained ferric ions at a concentration of 500 μM.

#### 2.3.2. Job’s Method

The standard Job’s method [35] was employed. In Job’s method, the concentration of both components (metal ions and a tested compound) are changing while their concentration sum is kept constant. This is based on the principle that the highest quantity of the complex will be formed under ideal conditions, which means that under such conditions the concentration ratio corresponds to the stoichiometry of the complex. On the contrary, in the conditions different from these ideal ones, there will be an excess of either the metal or the tested compound, which cannot form a complex since the other component in the reaction is missing. In our experiment, the molar concentrations of isoquercitrin + metal ion were kept constant at 100 µM while their molar concentration ratios were continuously changed from 1:3 to 6:1 throughout the series of samples. The measurements were performed against a blank composed of methanol and buffer in the ratio 1:2.

#### 2.3.3. Complementary Method

For the complementary approach the molar concentration of isoquercitrin was continuously changed, while the concentration of the metal was kept constant throughout the series of samples with different molar concentration ratios ranging from 1:3 to 6:1 (isoquercitrin:metal). The concentration of the metal was always 10 μM, while those of isoquercitrin was changing from 3.33 to 60 μM. The blank composition was the same as in the case of Job’s method. Calculation of the complex stoichiometry was based on our previously-mentioned mathematical approach [32]. In brief, molar absorption coefficients for both pure isoquercitrin and its complex with the particular metal ion were calculated by the measurements of a series of different concentrations of isoquercitrin without or with the metal (in its excess, see Section 2.3.1), respectively. Based on these coefficients, the theoretical lines mimicking the absorbance of the most probable stoichiometries were constructed and matched with real measured data of the abovementioned experiments with different ratios of isoquercitrin to a metal ion.

### 2.4. Competitive Measurement of Metal Chelation and Reduction

The general principle of these methods is the fact that indicators (BCS, hematoxylin or ferrozine) compete with isoquercitrin for the metal binding. Therefore, isoquercitrin was firstly mixed with a metal ion to allow the complex formation. After that, an indicator that is, in reality, also a metal chelator was added to the mixture. There are three possible scenarios depending on the metal and pH: (1) isoquercitrin is not chelating the metal at all or it binds very weakly; in that case the indicator will bind to all metal ions present in the solution; (2) isoquercitrin forms a very stable complex and the indicator is not able to remove it from the complex; or (3) isoquercitrin forms a moderately stable or unstable complex so the indicator can compete with isoquercitrin for the metal ions. In order to test the stability in both acute and delayed settings, the absorbance was measured immediately and after a certain period of time depending on the method. An unstable complex will lose its metal ion in time due to the formation of the complex indicator-metal thus the absorbance will be significantly higher at 5/7 min than immediately after mixing.

The methods can be used also for the assessment of reduction, since both BSC and ferrozine react only with the ions in lower valence (Cu^+^, Fe^2+^). Complexes of the indicator with respective metal ions are measured spectrophotometrically thereafter. All experiments were performed in 96-well microplates at room temperature (25 °C) and at least five different concentrations were measured. The detailed methodology was described in our previous papers [36,37] and it is described shortly hereunder.

#### 2.4.1. Ferrozine Method

Ferrozine is a specific indicator, which forms a magenta-colored complex with Fe^2+^ and it was, thus, used for the assessment of ferrous chelation at all the above-mentioned pH values. Additionally, total iron (Fe^2+^ + Fe^3+^) chelation at pH 4.5 was measured after reduction of ferric ions by HA. Under other pH conditions the reduction of ferric to ferrous ions was not complete and, thus, the methodology could not be employed for the precise assessment of total iron chelation [36]. However, the degree of reduction could be assessed at all pH conditions.

Various concentrations of isoquercitrin solutions in DMSO (50 μL) were mixed with the solution of ferrous or ferric ions (50 μL, 250 μM) in a buffer (150 μL) for 2 min. HA (50 μL, 10 mM) was added at pH 7.5 in order to inhibit ferrous oxidation at this pH. For the assessment of total iron chelation at pH 4.5, HA (50 μL, 10 mM) was used to reduce the remaining Fe^3+^ ions into Fe^2+^. Then, in all cases, ferrozine (50 μL, 5 mM) was added and the absorbance was measured immediately and 5 min later at 562 nm. For the determination of the degree of ferric ions reduction, the approach was similar, but HA was added only as the positive control (100% reduction).

#### 2.4.2. Hematoxylin Method

Hematoxylin specifically complexes cupric ions, which can also be measured spectrophotometrically. Isoquercitrin DMSO solutions (50 μL) at different concentrations were mixed with Cu^2+^ ions (50 μL, 250 μM) for 2 min in the presence of the respective buffer (150 μL). The mixture was incubated for a further 3 min with the indicator hematoxylin (50 μL, 250 μM) in order to enable the reaction of non-chelated copper ions with it. The absorbance was measured thereafter, and then after other 4 min (*i.e.*, at 7^th^ min). Different wavelengths were used according to pH: 595 nm (pH 5.5), 590 nm (pH 6.8), and 610 nm (pH 7.5), as given in our previous report. At pH 4.5, hematoxylin has apparently low affinity to cupric ions and hence the method cannot be employed [37].

#### 2.4.3. BCS Method

The BCS method is analogous to the ferrozine method, with the exception that BCS is specific to cuprous ions. The methodological approaches for both reduction and chelation were also almost identical with the exception of the use of BCS instead of ferrozine and the employment of HA (50 μL, 1 mM at pH 6.8/7.5 or 10 mM at pH 4.5/5.5), which was added in the case of all cuprous measurements. Detailed methodology can be found in our former study [37].

### 2.5. Statistical Analysis

The experiments were performed at least in duplicates with three different stock solutions. Data are shown as means ± SD. All statistical analyses were performed using the software GraphPad Prism version 6 for Windows (GraphPad Software, La Jolla, CA, USA).

## 3. Results

### 3.1. Determination of Complex Stoichiometry by Job’s and Complementary Methods

Firstly, the formation of complexes between the metal ions and isoquercitrin was tested at all pH values under non-competitive conditions, which means that isoquercitrin was mixed solely with the metal ion in a buffer. Addition of ferrous, ferric, and cupric ions to isoquercitrin at pH 5.5, 6.8, and 7.5 resulted in clear bathochromic shifts of the absorbance maxima from 355 nm to 404 or 421 nm depending on the metal (see Supplementary Material Table S1 and Figure S1). This suggests the formation of complexes between metal ions and isoquercitrin under these conditions. On the contrary, addition of Cu^+^ ions did not modify the absorbance spectrum of isoquercitrin revealing the inability of this flavonol to chelate cuprous ions. At pH 4.5, most metals behaved like cuprous ions, there were no spectral changes after addition of metal ions with the exception of ferric ions. Hence, at pH 4.5, isoquercitrin seems to form a complex with ferric, but not with ferrous or copper, ions (Table 1). There were generally no differences between the position of the maximum of isoquercitrin complexes with ferrous and ferric ions, implying the formation of an identical complex.

In the next step, molar absorption coefficients of the pure compound and the complexes formed were identified under the same non-competitive conditions. They are summarized in the Supplementary Material (Table S1). The coefficient of linear regression (R^2^) for multiple measurements (use of several new stock solutions) was higher than 0.97 in all cases.

**Figure 2:**
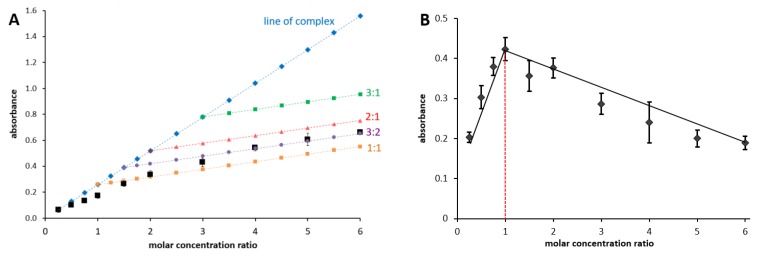
Assessment of the Fe^2+^-isoquercitrin complex stoichiometry at pH 6.8. Complementary approach (**A**): the final molar concentration of ferrous ions was 15 μM and the final molar concentration of isoquercitrin was 4–90 μM. The blue line corresponds to the absorbance of the formed complex at the excess of Fe^2+^ ions. Other lines show possible stoichiometries. Job’s plot (**B**): the total molar concentration of isoquercitrin and Fe^2+^ ions was 100 μM. In both cases, Fe^2+^ ions were allowed to react with isoquercitrin for 1 min before absorbance was measured at the maximum of the absorbance of the complex (λc, 404 nm). The molar concentration ratio signifies the ratio between the concentration of isoquercitrin to that of the ferrous ions. The assessment was performed with three independent stock solutions.

The last part of the non-competitive experiments concerned the determination of the complex stoichiometry. Two independent methods (Job’s and complementary methods) were employed. Under most conditions in which the metal complex was formed, the complex of the 1:1 stoichiometry was detected (Table 1, Supplementary Material Figure S2). There were, however, few exceptions: at pH 5.5, where the complex with Fe^2+^ ions was clearly formed, but the affinity of isoquercitrin toward Fe^2+^ was low and, therefore, both methods failed to establish the stoichiometry unambiguously. The isoquercitrin’s stoichiometry at pH 6.8 for Fe^2+^ was unusual: isoquercitrin was able to chelate iron at two different chelation ratios (1:1 and 3:2, isoquercitrin:iron, respectively) depending on its concentration (Figure 2A). On the other hand, the Job’s method showed only one ratio, 1:1 (Figure 2B).

### 3.2. Competitive Methods

**Figure 3:**
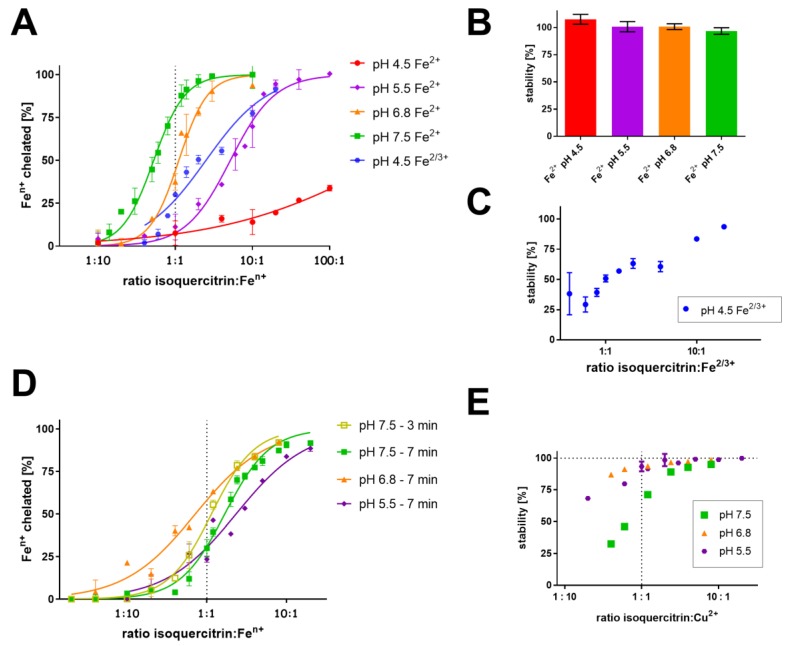
Competitive assessment of iron and copper chelation. Iron chelation by isoquercitrin (ferrozine method) is shown in section (**A**) with the stability of formed complexes in parts (**B**) and (**C**). Cupric chelation by isoquercitrin (haematoxylin method) is depicted in section (**D**); (**E**) describes the corresponding stability of these cupric complexes. Isoquercitrin was mixed with the respective metal ions in different buffers for 2 min, the indicator (ferrozine or haematoxylin) was added and the absorbance was measured immediately and then after 5 min (iron) or 7 min (copper, see the Experimental ection 2.4). The chelation results means the percentage of metal chelation calculated *vs.* blank sample containing the metal ions without isoquercitrin (data on iron are after 5 min, data on copper are specified in the legend). Stability was calculated as the change of percentage of metal chelation after 5/7 min *vs*. the first immediate measurement. Cupric chelation at pH 4.5 cannot be established due to low affinity of the indicator for copper at this pH.

In order to assess in more details the capacity of isoquercitrin to interact with metals, competitive measurements were performed. In these assays, the indicator competes with isoquercitrin for the chelated metal. This testing can determine if the complexes measured by the non-competitive method are also formed under competitive conditions and whether they are stable with respect to time. Here, in line with non-competitive measurement, isoquercitrin clearly chelated both iron (Fe^2+^ and Fe^3+^) and cupric ions (Figure 3). However, isoquercitrin chelation potency toward iron dropped with decreasing pH (Figure 3A). These measurements can also provide information on the probable stoichiometry of the complexes if the chelator is able to maintain at a certain condition the metal in competition with the indicator. The easiest way is to look at the ratio 1:1. For example, at pH 6.8, the curve of chelation intersects the 1:1 ratio line (x axis) at about 50% chelation (y axis), this suggests that at 1:1 ratio about 50% of iron is chelated, thus, the complex will have probably the 2:1, isoquercitrin:iron, stoichiometry. At pH 7.5, around 70% of ferrous ions is chelated at the ratio 1:1 and this evokes the 3:2 stoichiometry (isoquercitrin:iron). At pH 5.5 and 4.5, the stoichiometry cannot be clarified since the affinity of isoquercitrin toward iron was obviously low since the curve intersects the 1:1 line at low chelation (y axis) values. Ferrous complexes were stable in contrast to the ferric ones at pH 4.5 (Figure 3B,C).

Similar experiments were performed with copper ions (Figure 3D). Here, at mildly competitive conditions (hematoxylin method), the complexes with 2:1 stoichiometry (isoquercitrin:Cu^2+^) were apparently formed at pH 6.8 (See Figure 3D, at ratio 1:1 there is about 50% chelation). At pH 7.5, a complex with 2:1 stoichiometry was also formed but this complex was obviously not stable (Figure 3D,E).

**Figure 4:**
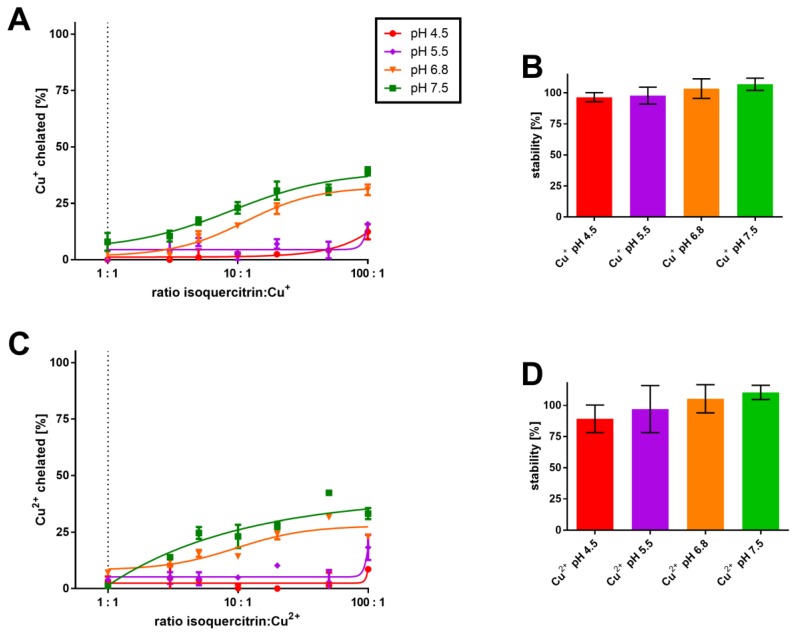
Copper chelation in highly competitive ambient (BCS method). (**A**) Cu^+^ chelation with corresponding complex stability (**B**); Cu^2+^ chelation (**C**) with respective complex stability (**D**). Isoquercitrin was mixed with copper ions (final concentration was kept the same in all experiments—50 μM) in different buffers for 2 min in the presence (cuprous ions) or absence of HA (cupric ions). HA was added thereafter in the case of Cu^2+^ ions in order to reduce non-chelated copper. In the last step, the indicator BCS was added. Absorbance was measured immediately and after 5 min. The chelation results means the percent of metal chelation calculated *vs*. the blank sample containing copper ions without isoquercitrin after a 5 min measurement. Stability was calculated as the change of percentage of copper chelation after 5 min *vs*. the first measurement at time 0.

In contrast, low chelation potency toward Cu^+^ and Cu^2+^ ions was shown under more competitive conditions in the BCS method. However, these complexes were very stable (Figure 4). Due to low potency, the stoichiometry cannot be assessed from these competitive data.

### 3.3. Iron and Copper Reduction

**Figure 5:**
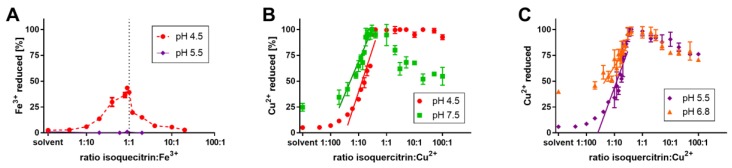
Metal reduction by isoquercitrin: (**A**) ferric ion reduction after 5 min. Data for pH 6.8 and 7.5 are not shown since there was no reduction similarly demonstrated for pH 5.5. (**B**,**C**) cupric reduction after 5 min. Isoquercitrin in respective buffers was mixed with Fe^3+^ or Cu^2+^ ions (the final concentration of both was 50 μM) and the indicator ferrozine or BCS was added, respectively. The absorbance was measured immediately and after 5 min. The percent reduction was calculated *vs*. positive control sample containing the Fe^3+^/Cu^2+^ ions with hydroxylamine as the reductant. There is a linear dependence between the reduction and ratio (or concentration) in the case of cupric reduction if we neglect both poles (maximal reduction and insignificant reduction *vs*. solvent).

As previously shown in numerous moderately-active chelators from the flavonoid class [25,31], isoquercitrin is able to reduce both cupric and ferric ions. Iron reduction was observed almost exclusively in the case of pH 4.5 and reached the maximum of 35% in the proximity of the ratio 1:1, while with increasing concentrations the reducing power dropped (Figure 5) in line with the above described chelation ability of isoquercitrin. Additional experiments in time showed that iron reduction can also take place, although at a much smaller scale, at pH 5.5 after longer incubation, but not at higher pH (Supplementary Figure S3). In the case of cupric ions, isoquercitrin was a very active reductant and was able to reduce 100% of copper after 5 min of incubation at all pH conditions tested (Figure 5). In higher ratios of isoquercitrin over copper, some decrease of copper reduction was also observed, in particular in higher pH conditions, again in line with its chelation ability (Figure 3D). Supplementary experiments in time showed that, again, this reduction process is increasing in time (Supplementary Figure S4).

## 4. Discussion

Flavonoids have been attracting much interest due to their various, potentially beneficial effects on human health [38,39]. In the present series of experiments we selected the glycosylated flavonol isoquercitrin as a suitable model compound with moderate affinity for metals, in order to perform the assessment of its behavior *versus* both mentioned transition metals. To the best of our knowledge, metal interactions of isoquercitrin were tested only with aluminum and marginally with iron [22,23,24].

For aluminum, three possible complexes were formed with the stoichiometries 1:1, 2:1, and 1:2, isoquercitrin:Al^3+^. However, the authors stated that the third stoichiometry was formed only under specific conditions (high excess of Al^3+^) [22]. Two other studies showed, by a spectral shift, that Fe^2+^ formed a complex with isoquercitrin, but no attempts for stoichiometry elucidation were performed [23,24].

The molecules of flavonoids consist of two phenyl rings (A and B) and one heterocyclic ring (C, also shown in Figure 1). There is a general agreement that possible chelation sites of flavonoids include two proximal hydroxyl groups (*o*-dihydroxy group in ring B or ring A) or 3-hydroxy-4-keto or 5-hydroxy-4-keto moieties [40,41]. The selection of isoquercitrin for the present study was intentional, since it does not contain the chelation site in ring A, and also another possible chelation site in flavonols in ring C is unavailable due to the substitution of the 3-hydroxy group by glucose (Figure 1). Thus, there exist two possible chelation sites - catechol ring B and 5-hydroxy-4-keto moiety. We have shown in our previous experiments that 5-hydroxy-4-keto moiety has generally low affinity for metals [40] and as the lowest chelation ratio observed in this study was 1:1, we suggest that one chelation site was likely employed in the chelation of copper or iron in most cases. Interestingly, this does not seem to be true for Al^3+^, where both the 3′,4′-dihydroxyl group and also the 5-hydroxy-4-keto moiety likely participated in the chelation [22]. In the present study, sufficient chelation in competitive conditions were observed only in the case of neutral or slightly acidic pH. Based on this pH dependence, this chelation site of isoquercitrin is probably at the 3′,4′-dihydroxyl group in most conditions. Indeed, the catechol ring B is the obvious effective chelation site at neutral conditions but it has only low activity at more acidic pH [40].

In general, the 3-hydroxy-4-keto moiety is strongly involved in chelation and this can be confirmed by direct comparison of chelation potency of isoquercitrin (glycosylated at C-3) and that of quercetin having this moiety available for chelation. To date there has been no study directly comparing chelation potency of these related flavonols, however, we can compare the data in this paper with our previous studies, which were performed using the same methodical approach [28,40]. Indeed, quercetin is a more potent chelator of copper and iron, with an exception of more acidic pH (4.5 and 5.5), where there is no significant difference in the potency between both compounds (Supplementary Figure S5). In addition, quercetin is an unambiguously stronger chelator of cuprous ions [28] than isoquercitrin (Figure 4).

The complex 1:1 of isoquercitrin to metal was formed under most conditions with an important exception. Under the mild acidic conditions (pH 6.8), isoquercitrin chelated ferrous ions at two chelation ratios 1:1 and 3:2 (isoquercitrin to iron), depending on its concentration. On the other hand, the Job’s method showed only one ratio—1:1. This seems to be a discrepancy at first sight, but we suppose that both methodologies are additive. The difference can be simply explained by the different methodological approach, consisting of stable or changing metal concentrations and the ensuing methods’ limitations. Job’s method can establish the stoichiometry kinetics only if the stoichiometry is changing gradually. However, if the different complex is formed only in a clear excess of the chelator over the metal, the complementary approach seems to be more advantageous [32]. We have previously shown an analogous change of the complex stoichiometry by our complementary approach for a very similar molecule - rutin [32]. The chemical difference between rutin and isoquercitrin is only one sugar entity (α-l-rhamnose) attached at C-6″ of the glucosyl moiety. Importantly, the ideal 1:1 stoichiometry of the isoquercitrin-metal complex is also changing in competitive settings, when an indicator with a good affinity to the metal is present. This was demonstrated for both copper and iron, since one molecule of isoquercitrin was unable to chelate one atom of the metal even at neutral conditions. This clearly suggests that complexes with the stoichiometry of 1:1 formed at non-competitive conditions are not stable. This study, thus, brings an important secondary outcome - the necessity to perform different approaches for the characterization of the complex stoichiometry in the case of moderate or weak chelators, like most flavonoids. Indeed, we suppose that the use of Job’s method in combination with other methods is advantageous and the results are not contradictory; rather, they might bring more complex information about the kinetics and stability of metal chelation. This finding may also help to explain the different results with flavonoids from previous studies [42,43].

The present work was not aimed at the determination of the complex structure, however, one plausible conclusion can be made. Since there were no apparent differences in the absorption maxima between Fe^2+^ and Fe^3+^ complexes, probably only a single type of iron-isoquercitrin complex was formed. Since ferrous ions may be oxidized in the complexes with iron chelators under physiological pH conditions and in the presence of oxygen, ferric complexes were likely formed [44], but we do not have experimental data to confirm it.

The biological relevance is difficult to establish from this study since isoquercitrin was also shown to be a potent reducing agent with the ability to reduce both copper and iron. In addition, the influence of pH is inverse when comparing iron to copper. In the case of copper, increasing pH from acidic to neutral increases the reduction potential, whilst in the case of iron, decreasing pH increases the reduction potential. Hence, its systemic clinical use as a therapeutic agent is not plausible. On the other hand, it should be mentioned that isoquercitrin is a frequent component of human diet (fruits, vegetables, cereals and, more importantly, food supplements), which typically provides tens of mg/day and can thus influence iron and copper absorption in the gastrointestinal tract [45]. There are almost no data for flavonoid interaction with metals in the gastrointestinal tract, but it has been demonstrated that tea or coffee decreased iron and lactovegetarian diet copper absorption from the gastrointestinal tract [46,47]. Interestingly, the flavanol catechin did not interact with iron absorption and the authors from the first study explained it was due to its low water solubility. Therefore, the interaction of more hydrophilic isoquercitrin should be tested at this level in the future. Simple biochemical data cannot, at the moment, sufficiently predict the interaction, since flavonoids can reduce ferric ions into ferrous ions and, thus, enable their absorption or possibly some flavonoid-metal complexes can be absorbed better than pure flavonoids. Additionally, the biological behavior of isoquercitrin must be considered. Data on rutin are more frequently reported, but even when the chelation/reduction effects and the spectrophotometric behavior are similar, there are some differences in physical, chemical, and biological properties between isoquercitrin and rutin [1,48]. For example, their aglycon, quercetin, is more bioavailable from isoquercitrin [49], which is also more water soluble [50] and has a more potent antiproliferative effect than rutin [10]. In addition, there can also be a slight, but important, difference between the behavior of isoquercitrin and rutin towards iron and copper ions [23]. This suggests that whenever the in vitro behavior of quercetin glycosides is similar, in vivo evaluation of isoquercitrin chelation potency in the context of the whole organism is badly needed.

## 5. Conclusions

This study confirmed that isoquercitrin is a moderately-active ferrous, ferric, and cupric chelator, in particular under neutral or slightly acidic pH conditions, which is *i.a.* relevant for resorption of these metals in digestive tract. In line with this, the most plausible chelation site is the 3′,4′-dihydroxyl (catechol) moiety. Stoichiometry of respective metal complexes is typically 1:1 under ideal conditions, but changes in excess isoquercitrin or under competitive conditions. This study also brought a secondary outcome—the necessity to use multiple methodologies in order to better establish the chelation behavior of moderately-active metal chelators.

## Figures and Tables

**Table 1:** Summarized results of the formation and stoichiometry of isoquercitrin complexes with iron and copper at all pH values analyzed.

		pH 4.5	pH 5.5	pH 6.8	pH 7.5
	Job’s method	1:1	1:1	1:1	1:1
Fe^3+^	Complementary approach	1:1	1:1	1:1	1:1
	Competitive method	low affinity	X	X	X
	Job’s method	no complex	low affinity	1:1	1:1
Fe^2+^	Complementary approach	no complex	low affinity	1:1 → 3:2	1:1
	Competitive method	low affinity	low affinity	2:1	3:2
	Job’s method	no complex	1:1 or 3:2	1:1	1:1
Cu^2+^	Complementary approach	no complex	1:1	1:1	1:1
	Competitive method	X	low affinity	2:1	2:1 *
	Job’s method	no complex			
Cu^+^	Complementary approach	no complex			
	Competitive method	low affinity			

The data are shown as a ratio of isoquercitrin to metal. Low affinity—the complex was detected but due to low affinity of isoquercitrin toward the metal, the stoichiometry cannot be determined; *—unstable; X—method cannot be applied.

## Supplementary Materials

The supplementary file is available online at www.mdpi.com/2072-6643/9/11/1193/s1. Table S1: Summarized data on absorption maxima and molar absorption coefficients of isoquercitrin and its complexes with iron and copper, Figure S1: Illustrative examples of absorption spectra of isoquercitrin alone and with different metals. Isoquercitrin and Fe2+ pH 6.8 (A). Isoquercitrin and Fe3+ pH 7.5 (B). Isoquercitrin and Fe2+ pH 4.5. No complex is formed (C). Isoquercetrin and Cu2+ pH 4.5. No complex is formed (D). Isoquercitrin and Cu2+ pH 6.8 (E). Isoquercitrin and Cu+ pH 7.5. No complex is formed (F), Figure S2: Illustrative examples of the determination of isoquercitrin-metal stoichiometry, Figure S3: Kinetics of iron reduction by isoquercetrin, Figure S4: Kinetics of copper reduction by isoquercetrin, Figure S5: Comparison of chelation potential between quercetin and isoquercitrin.

## Abbreviations

The following abbreviations are used in this manuscript:

BCS	bathocuproinedisulphonic acid disodium salt
HEPES	4-(2-hydroxyethyl)-1-piperazineethanesulphonic acid
HA	hydroxylamine hydrochloride
