# Supplementary material for: The Stoichiometry of Isoquercitrin Complex with Iron or Copper Is Highly Dependent on Experimental Conditions
Source: Nutrients. 2017 Oct 30;9(11):1193. doi: 10.3390/nu9111193 (PMC5707665; doi:10.3390/nu9111193)
Supplement: Supplementary file 1 [file nutrients-09-01193-s001.pdf]

Supplementary Materials: The Stoichiometry of Isoquercitrin Complex with Iron or Copper Is Highly Dependent on Experimental Conditions

Maria Carmen Catapano[+], **Václav Tvrdý**[+], **Jana Karlíčková**[++], **Thomas Migkos**[+], **Kateřina Valentová**[#], **Vladimír Křen**[#] and **Přemysl Mladěnka**[+*]

Table S1. Summarized data on absorption maxima and molar absorption coefficients of isoquercitrin and its complexes with iron and copper.

	**pH 4.5**	**pH 5.5**	**pH 6.8**	**pH 7.5**
λ_S	353 ± 1	354 ± 0.6	355 ± 1	356 ± 2
λ_C (Fe^{2+}) [nm]	no complex		404 ± 1	
λ_C (Fe^{3+}) [nm]			404 ± 1	
λ_C (Cu^{2+}) [nm]	no complex		421 ± 2	
λ_C (Cu^{+}) [nm]	no complex			
ε_S [$mol^{-1} \cdot cm^{-1} \cdot l$) × 10^3	14 ± 0.4 (0.9867)	19 ± 0.8 (0.9703)	18 ± 0.6 (0.9803)	16 ± 0.3 (0.9947)
ε_C (Fe^{2+}) [$mol^{-1} \cdot cm^{-1} \cdot l$] × 10^3	no complex	10 ± 0.4 (0.9717)	15 ± 0.3 (0.9883)	13 ± 0.3 (0.9994)
ε_C (Fe^{3+}) [$mol^{-1} \cdot cm^{-1} \cdot l$] × 10^3	19 ± 0.5 (0.9976)	18 ± 0.5 (0.9975)	12 ± 0.2 (0.9996)	18 ± 0.5 (0.9973)
ε_C (Cu^{2+}) [$mol^{-1} \cdot cm^{-1} \cdot l$] × 10^3	no complex	19 ± 0.5 (0.9979)	16 ± 0.1 (0.9999)	17 ± 0.4 (0.9982)

λ_S: absorbance maximum of the substance (isoquercitrin).
λ_C: absorbance maximum of the complex.
ε_S: molar absorption coefficient of substance at the maximum.
ε_C: molar absorption coefficient of complex at the maximum.

N.B. Molar absorption coefficients of the substance (ε_s) and of the complex (ε_c) were calculated according to the Lambert–Beer law.

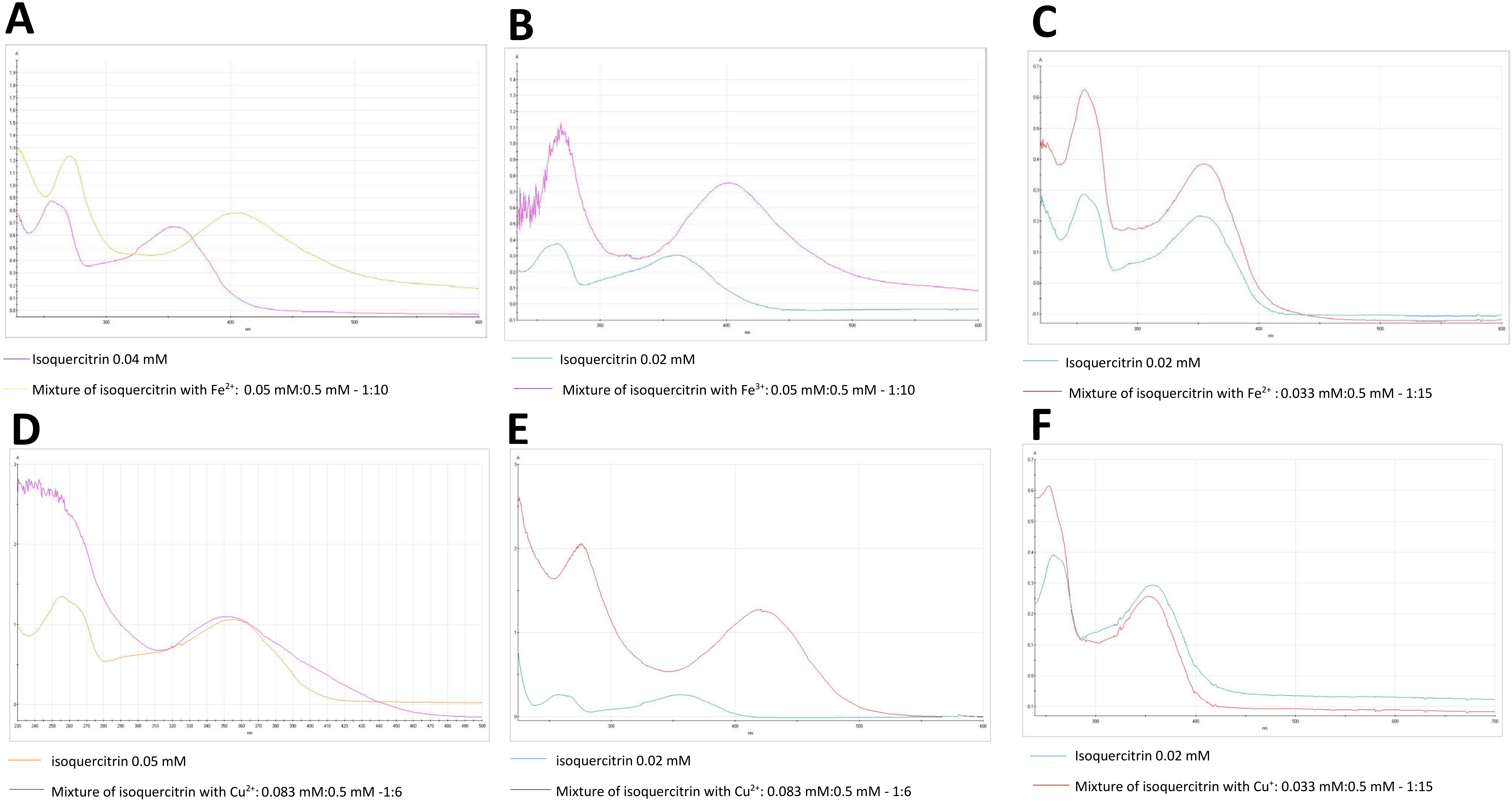

Figure S1. Illustrative examples of absorption spectra of isoquercitrin alone and with different metals. Isoquercitrin and Fe^{2+} pH 6.8 (**A**). Isoquercitrin and Fe^{3+} pH 7.5 (**B**). Isoquercitrin and Fe^{2+} pH 4.5. No complex is formed (**C**). Isoquercetrin and Cu^{2+} pH 4.5. No complex is formed (**D**). Isoquercitrin and Cu^{2+} pH 6.8 (**E**). Isoquercitrin and Cu^{+} pH 7.5. No complex is formed (**F**). The absorption spectra ranged from 220 to 800 nm. Wavelength(s) of absorption maximum(a) of a tested substance (λ_{Smax}) were determined at all pH values. Similarly, a determination of the wavelength(s) of absorption maximum(a) of the complex (λ_{Cmax}) were accomplished by the use of metal excess at different concentration ratios ranging from 1:6 to 1:50 (substance:metal ion). The blank was composed of the buffer, methanol and water in the ratio 1:1:1 for isoquecitrin and isoquercitrin+Fe^{2+} measurement while, for the ferric complex, the blank contained in addition 500 μM of ferric ions.

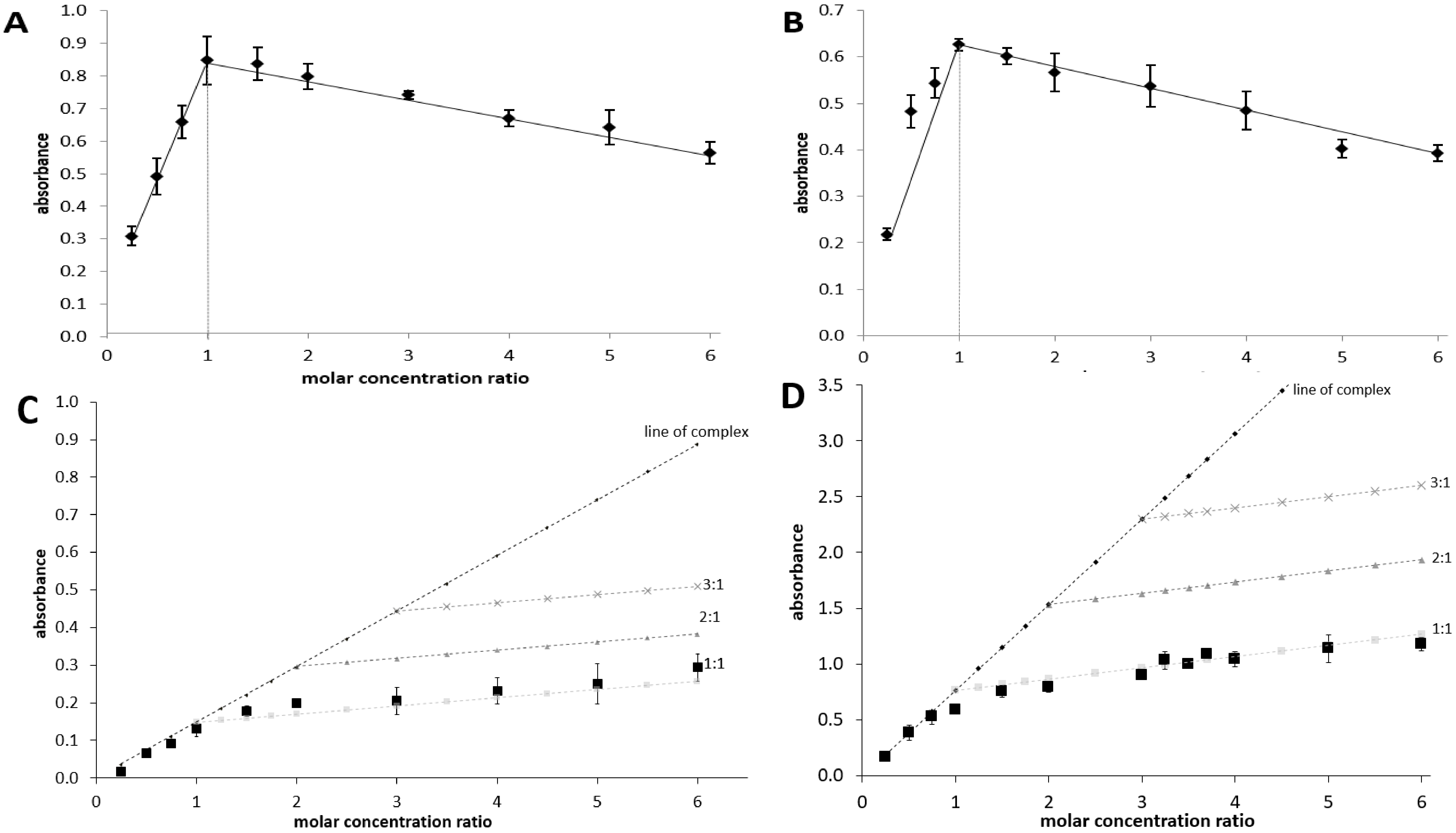

Figure S2. Illustrative examples of the determination of isoquercitrin-metal stoichiometry. Data for pH 7.5 and ferrous ions are showed at the left side: (**A**) (Job's method) and (**C**) (complementary method). Analogously for pH 7.5 and ferric ions are showed at the right side: (**B**) (Job's method) and (**D**) (complementary method). Absorbance was measured in both conditions at λc (404 nm). The molar concentration ratio is the ratio between the concentration of isoquercitrin to the metal ions. The total molar concentration of isoquercitrin and iron was 100 μM regarding Job's method for both ferric and ferrous ions. In the case of the complementary approach, the final molar concentration of iron (Fe^{2+}, Fe^{3+}) was 10 μM and the final molar concentration of isoquercitrin was gradually changed from 5 to 60 μM. The assessment was performed with two different stock solutions.

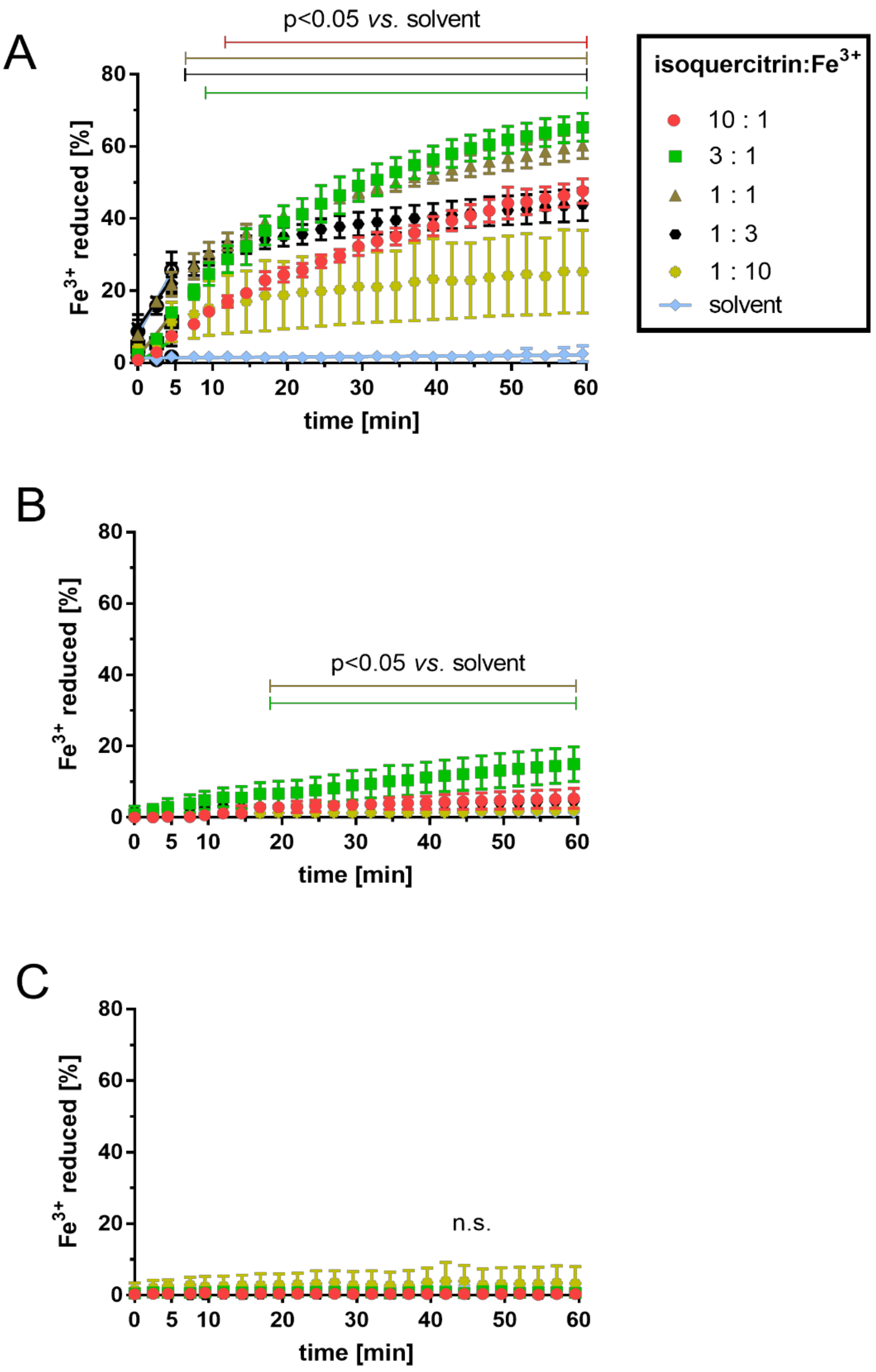

Figure S3. Kinetics of iron reduction by isoquercetrin. Isoquercetrin was incubated up to 60 minutes with ferric ions (final concentration 50 µM) in different ratios together with the indicator, ferrozine: (**A**) pH 4.5; (**B**): pH 5.5; and (**C**) pH 6.8. The percent of reduction is related to the positive control—maximal iron reduction was caused by the addition of hydroxylamine. Statistical significance vs. solvent is shown. At pH 6.8, no significant reduction was observed while, in the case of pH 5.5, significance was detected only for ratios 1:1 and 3:1, isoquercetrin to iron, from min 17 to min 60. The relationship reduction on time seems to be linear at least within the first five minutes (lines are shown in the figures, in particular at pH 4.5).

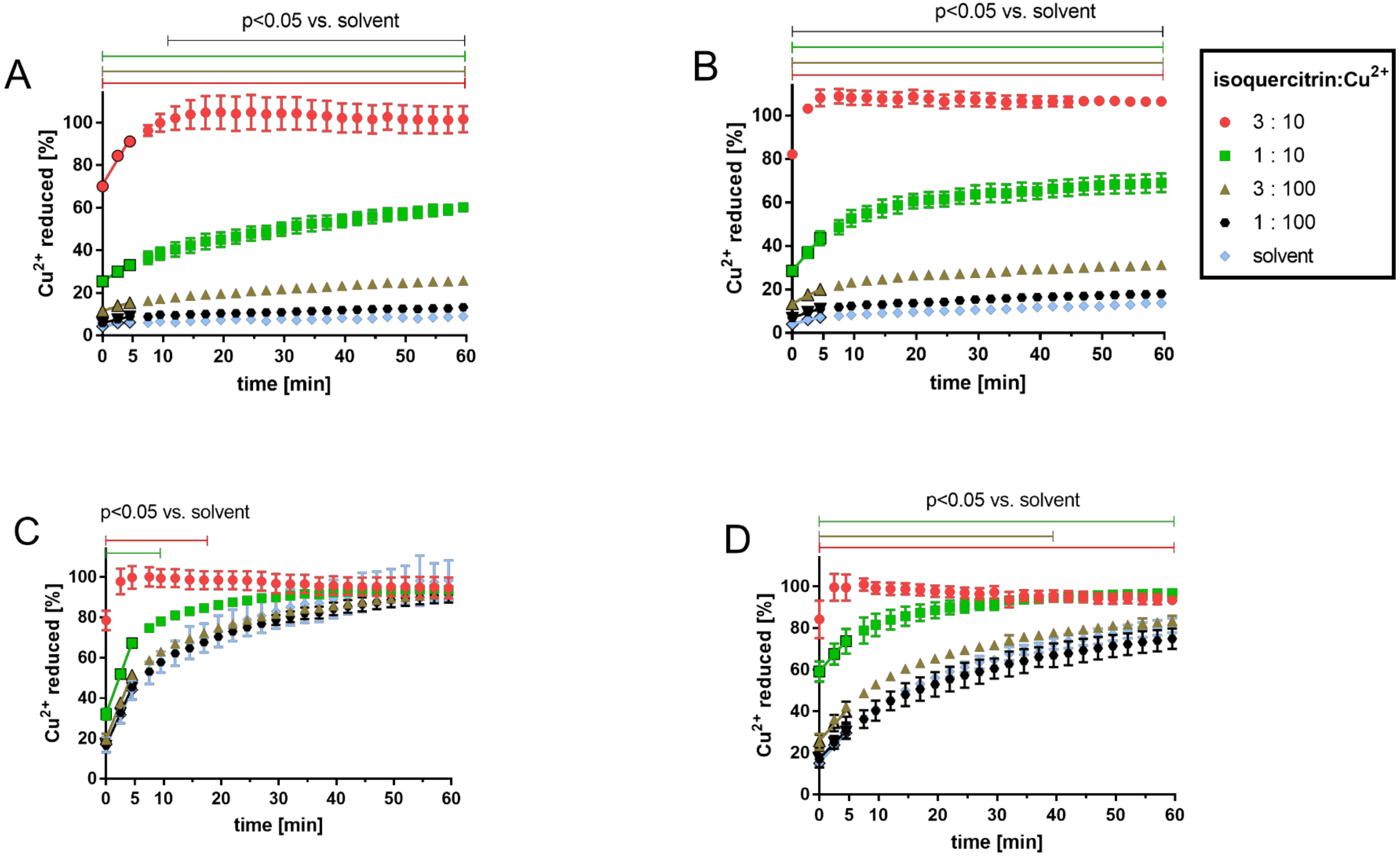

Figure S4. Kinetics of copper reduction by isoquercetrin. Isoquercetrin was incubated up to 60 minutes with cupric ions (final concentration 50 μM) in different ratios together with the indicator, BCS: (**A**): pH 4.5; (**B**) pH 5.5; (**C**) pH 6.8; and (**D**) pH 7.5. The percent of reduction is related to the positive control—maximal copper reduction was caused by addition of hydroxylamine. Statistical significance vs. solvent is shown. The relationship reduction on time seems to be linear at least within the first five minutes (lines are shown in the figures), such relationship was not found for the highest tested ratio (3:10) since, apparently, the maximal reduction was reached earlier than at five minutes.

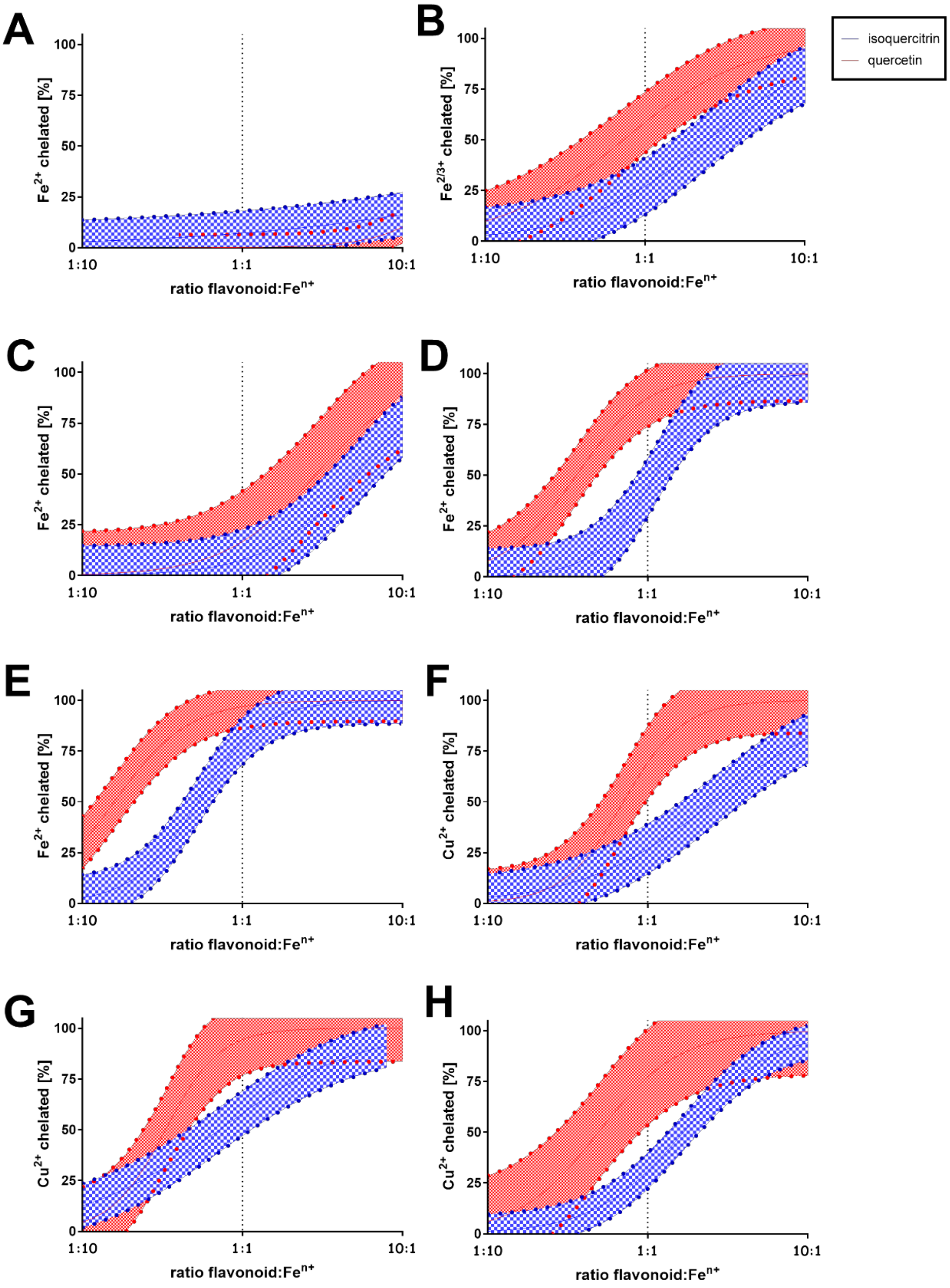

Figure S5. Comparison of chelation potential between quercetin and isoquercitrin. The graphs show 95% prediction bands of chelation. (**A**) pH 4.5, Fe^{2+}; (**B**) pH 4.5, $Fe^{2/3+}$; (**C**) pH 5.5, Fe^{2+}; (**D**) pH 6.8, Fe^{2+}; (**E**) pH 7.5, Fe^{2+}; (**F**) pH 5.5, Cu^{2+} (HEM); (**G**) pH 6.5, Cu^{2+} (HEM); and (**H**) pH 7.5, Cu^{2+} (HEM). Data for the calculation of prediction bands for quercetin were re-used from our previous articles [28,40]. There is a clear overlap at pH 4.5 (A) and 5.5 (C) for ferrous chelation, suggesting the same potency of both related flavonols at these conditions. In other cases, quercetin is a clearly more potent Cu/Fe chelator.
